# Detection of Carcinoembryonic Antigens Using a Surface Plasmon Resonance Biosensor

**DOI:** 10.3390/s8074282

**Published:** 2008-07-18

**Authors:** Fengyu Su, Chunye Xu, Minoru Taya, Kimie Murayama, Yasuro Shinohara, Shin-Ichiro Nishimura

**Affiliations:** 1 Center for Intelligent Materials and Systems, University of Washington, Box 352600, Seattle, WA98195, USA; 2 Division of Biomembrane Research, Pacific Northwest Research Institute, Seattle, WA98119, USA; 3 Laboratory of Advanced Chemical Biology, Graduate School of Advanced Life Science, Hokkaido University, Sapporo 001-0021, Japan

**Keywords:** Surface plasmon resonance (SPR), early detection of cancer, carcinoembryonic antigen (CEA)

## Abstract

Carcinoembryonic antigen (CEA) is an oncofoetal cell-surface glycoprotein that serves as an important tumor marker for colorectal and some other carcinomas. In this work, a CEA immunoassay using a surface plasmon resonance (SPR) biosensor has been developed. SPR could provide label-free, real-time detection with high sensitivity, though its ability to detect CEA in human serum was highly dependent on the analytical conditions employed. We investigated the influences of various analytical conditions including immobilization methods for anti-CEA antibody and composition of sensor surface on the selective and sensitive detection of CEA. The results show that anti-CEA antibody immobilized via Protein A or Protein G caused a large increase in the resonance signal upon injection of human serum due to the interactions with IgGs in serum, while direct covalent immobilization of anti-CEA antibody could substantially reduce it. An optimized protocol based on further kinetic analysis and the use of 2^nd^ and 3^rd^ antibodies for the sandwich assay allowed detecting spiked CEA in human serum as low as 25 ng/mL. Furthermore, a self-assembled monolayer of mixed ethylene-glycol terminated alkanethiols on gold was found to have a comparable ability in detecting CEA as CM5 with thick dextran matrix and C1 with short flat layer on gold.

## 1. Introduction

Diagnosis at an early stage and prognosis for successful therapeutic intervention are essential for the recovery of a cancer patient. Monitoring cancer biomarkers in blood, urine and other body fluids is an important method for early detection, since some of the biomarkers allow for identification of a disease at its very early stage, even before its symptoms can be recognized by a patient [1].

Carcinoembryonic antigen (CEA) is a widely used tumor marker for diagnostic and therapeutic purposes in gastrointestinal, breast and lung cancer [2]. The normal range for CEA in an adult non- smoker is less than 2.5 ng/mL and for a smoker less than 5.0 ng/mL in human serum, but its level exceeds 100 ng/mL upon development of certain cancers [3]. A rising CEA level indicates progression or recurrence of the cancer, which indicates detection of CEA in human sera samples can be used to diagnose and monitor cancer at its early stage. We have recently analyzed the glycoforms on human CEA purified from liver metastases of colon carcinoma and pleural and ascites fluids, and quantitatively detected more than 120 glycans on human CEA [4]. Substantially different glycosylation profiles between CEAs of liver metastases of colon carcinoma and pleural and ascites fluids were observed. In this regard, it will be very important not only quantify CEA level in serum but also quantitatively evaluate glycoforms attached for the improved sensitivity and specificity of diagnosis and prognosis. Indeed, although prostate-specific antigen (PSA) tests often suffer from lack of specificity in distinguishing benign prostate hyperplasia from prostate cancer, recent studies indicate that *N*-glycans of PSA found in prostate cancer differ significantly from those seen in benign prostate hyperplasia and therefore could be a potential indicator leading to improved sensitivity in diagnosing prostate cancer [5].

The majority of techniques currently employed to detect interactions of antigens and antibodies require a fluorescent- or enzymatic-labeling to report binding event [6], which is time-consuming, and may not be a suitable platform for the successive interaction analysis to reveal both the CEA level and the glycoform attached. Since surface plasmon resonance (SPR) can provide label-free and real-time detection, it has attracted strong attention as a biosensor in the past decade [7-9]. Biosensors based on SPR have been extensively used to monitor molecular interactions for its outstanding sensitivity, reliability, reproducibility as well as its capability to monitor multiple interactions successively [10, 11]. However, its application to the screening of disease markers in human body fluids (e.g. serum) is so far limited. To the best of our knowledge, the detection of the cancer marker CEA in human serum using a SPR biosensor is reported here for the first time.

In this paper, CEA was detected as an analyte in solution through its interactions with anti-CEA antibodies as ligands immobilized on the surface of a sensor chip. The most commonly used sensor chip consists of a carboxymethyl (CM) dextran matrix on the gold substrate [12]. The carboxylic groups of the dextran layer can be activated and then covalently coupled with the amino groups of proteins [13,14]. Besides the dextran matrix, self-assembled monolayers (SAMs) of alkanethiols have been introduced as immobilization layers [15, 16]. For SPR biosensing, Frederix *et al.* previously demonstrated that compared to a dextran layer a SAM is more advantageous for detection of low concentrations of analyte [17]. In this study, three kinds of sensor chips were evaulated, CM5 with a dextran matrix, C1 without a dextran matrix, and SAM of (1-mercapto-11-undecyl)tetra(ethylene glycol) (HSC_11_EG_4_OH) and (1-mercapto-11-undecyl)hexa(ethylene glycol)carboxylic acid (HSC_11_EG_6_OCH_2_COOH) on gold film for detecting CEA.

Since the amount and orientation of anti-CEA antibodies on the sensor chip surface are important factors related to the SPR sensitivity, direct covalent binding and indirect binding via protein A, protein G and anti-mouse polyclonal antibodies for the immobilization of anti-CEA antibodies were compared. Furthermore, a sandwich assay was performed by applying second and third antibodies to increase the SPR sensitivity and the possibility to detect antigens of even smaller amount in serum sample.

## 2. Results and Discussion

### 2.1 Effect of immobilization method on the detection of CEA

Antibodies could be immobilized directly on the sensor surface or indirectly to the surface through binding to another immobilized molecule that is called a capturing molecule. The direct immobilization is a covalent binding of amine group of antibody with carboxylic group on the sensor chip. The advantage of the direct binding is that antibody molecules are close to the sensor chip, which is favorable for higher sensitivity, while the disadvantage is the orientation of the antibody molecules on the sensor surface being random. The indirect immobilization includes covalent binding of capturing molecules with the sensor chip and molecular interaction of capturing molecules with the antibodies. In this work, we used anti-mouse rabbit IgG polyclonal antibody, Protein A and Protein G as capturing molecules. Immobilization via Protein A and Protein G should allow most of the Fab portions of the immobilized anti-CEA IgG accessible to the antigen (CEA) [18, 19].

**Figure 1.**
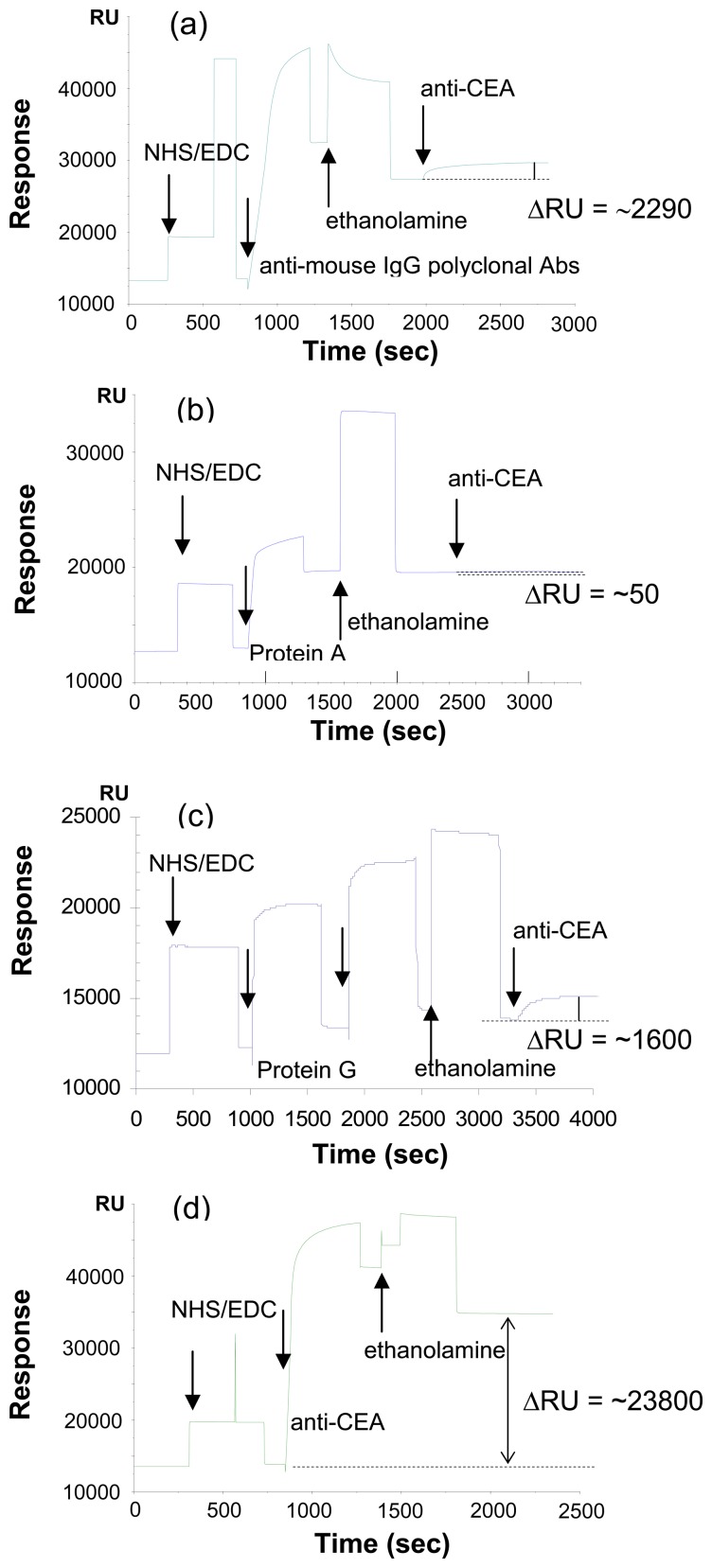
Immobilization of anti-CEA antibody onto sensor chip CM5; (a) via anti- mouse IgG polyclonal antibody, (b) via Protein A, (c) via Protein G, and (d) direct covalent binding.

Figure 1 shows the sensorgrams of immobilizing anti-CEA antibodies onto CM5 sensor chip via four different routes: (a) anti-mouse IgG polyclonal antibody, (b) Protein A, (c) Protein G and (d) direct covalent binding. The change in the resonance unit is shown on the *y*-axis, with the time of the reaction on the *x*-axis. The immobilized amounts of anti-CEA antibody via the four methods are 2,290, 50, 1,600 and 23,800 respectively. This indicates the direct covalent binding adsorb more anti-CEA antibodies onto the sensor chip, and anti-mouse polyclonal antibody and Protein G surfaces adsorb reasonable amount of anti-CEA antibodies, while these via Protein A are found to adsorb few amount of anti-CEA antibodies.

Immobilization via Protein A and Protein G are expected to provide well-oriented anti-CEA antibodies, which is beneficial for the further detection of antibody and antigen interactions. Considering that the introduced anti-CEA antibody belongs to IgG1 subclass, the extremely low capacity of Protein A should be attributed to its weak affinity to IgG1.

**Figure 2.**
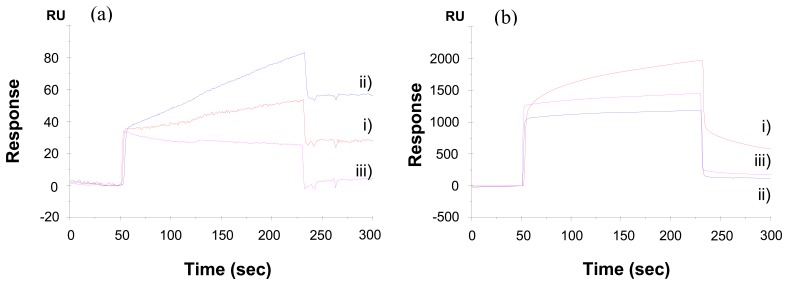
Sensorgrams of detecting (a) 100 ng/mL of CEA in HBS buffer and (b) 10-fold diluted human serum in HBS buffer on the three kinds of surfaces: i) via anti-mouse IgG polyclonal antibodies, ii) direct covalent immobilization, and iii) unmodified reference surface.

Interactions of CEA with anti-CEA antibody immobilized surface and control dextran surface are shown in Figure 2(a). The resonance unit change on the two kinds of surfaces in response to the analyte solution of 100 ng/mL of CEA in HBS buffer was 56 and 23 RU for direct binding surface, and the anti-mouse polyclonal antibody, respectively. Considering that the amount of antibody immobilized onto the sensor chip via anti-mouse IgG polyclonal antibody is only one tenth of that via direct binding (resonance unit shift 2,290 to 23,800), and further signal shift to interact with antigen is two fifth (resonance unit shift 23 to 56), it is highly possible the antibody on anti-mouse IgG polyclonal antibody has better orientation, and also the crowded antibodies on both surfaces limit the binding capacity. As a result, the direct covalent binding immobilizes much more of the anti-CEA onto the surface, and shows higher sensitivity to the antigen solution.

The susceptivity to non-specific bindings of the two kinds of surfaces is shown in Figure 2(b). Human serum diluted 10-fold in HBS buffer caused a high signal shift of around 600 RU on the sensor chip of anti-CEA on anti-mouse IgG polyclonal antibody, and a low signal shift on the anti-CEA surface through direct covalently binding.

The fact that the polyclonal IgGs surface gave a substantially high resonance unit increase is most probably due to the cross-reaction between immobilized anti-mouse antibodies and human IgGs present in serum in extremely high concentration. Likewise, anti-CEA antibody surfaces constructed through Protein A and Protein G immobilization also gave substantial resonance signal increases upon injection of human serum (data not shown). Considering that protein A and Protein G bind to IgGs of many different species including human, its application for the immunoassay using human serum is obviously problematic, since IgG is the second abundant protein in human serum with its average concentration in adult reaches as high as 12 mg/mL. From this point of view, covalent immobilization was found to be more advantageous for the immobilization of anti-CEA than indirect immobilization via rabbit polyclonal IgGs, Protein A and Protein G for the detection of CEA in serum sample, since it showed high response to CEA and low response to human serum.

For regeneration of the sensor surface, glycine·.HCl (pH 1.5) was used after antibody-antigen interactions. The antigen-antibody pair on the sensor surface through direct binding was broken, leaving antibody on the surface for use in further detections. In contrast, non-covalently immobilized anti-CEA antibody via anti-mouse IgG polyclonal antibody, Protein A and Protein G was removed from the surface by the regeneration process using glycine·HCl (pH 1.5), so the introduction of anti- CEA antibody is required for each analysis, which takes more steps. Considering the facts above, we decided to use direct covalent binding for the further study.

### 2.2. Kinetic analysis of interactions of carcinoembryonic antigens and antibodies

Kinetic analysis should provide important parameters in designing the experiment conditions to improve the detection sensitivity. Therefore, the interaction of CEA with immobilized anti-CEA antibody was analyzed under a series of concentration (12.5 ng/mL ∼ 800 ng/mL). The kinetic parameters were calculated based on the global analysis of the experimental data using a 1: 1 binding model accounting for mass transport.

**Figure 3.**
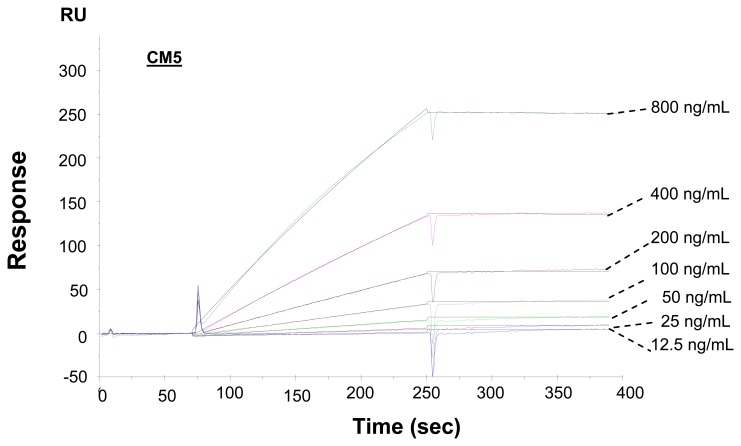
Kinetic analysis of CEA antibody and antigen interactions, detection of CEA antigens with a series of concentrations (800 ng/mL to 12.5 ng/mL): color lines are experimental sensorgrams, and black lines are simulated sensorgrams by using BIAevaluation 4.1.

**Figure 4.**
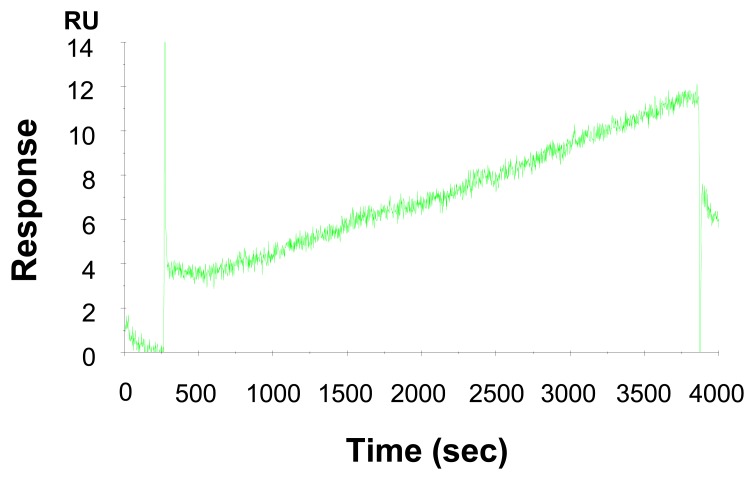
Sensorgram of detecting 6.2 ng/mL of CEA in HBS buffer.

The association rate constant (*k*_a_), dissociation rate constant (*k*_d_) and equilibrium constant (*K*_D_) were calculated to be 4.22 × 10^5^, 3.0 × 10^-5^, and 7.11 × 10^-11^, respectively. Based on the kinetic parameters, the change of resonance unit with the interaction time to the analyte solutions could be simulated by using the BIA evaluation software. As shown in Figure 3, the experimental data and the simulated data of the interactions of immobilized anti-CEA antibodies with antigens of a series of concentrations agree very well. For a low CEA in HBS concentration of 6.25 ng/mL, the simulation indicates that the resonance unit increase is less than 1 RU in 300 seconds, while it could reach to 10 RU in 3600 seconds, which is a change from undetectable to a detectable range. Consequently the interaction time was extended to 3600 seconds, and the resonance unit shift was 8 RU, as seen in the experimental sensorgram of Figure 4. This is quite similar to the simulated result of 10 RU. The above result indicates that even lower concentration of CEA (e.g. ∼1 ng/mL) can be detectable by extending the interaction time.

### 2.3. Method to increase the sensitivity --- sandwich assay

**Figure 5.**
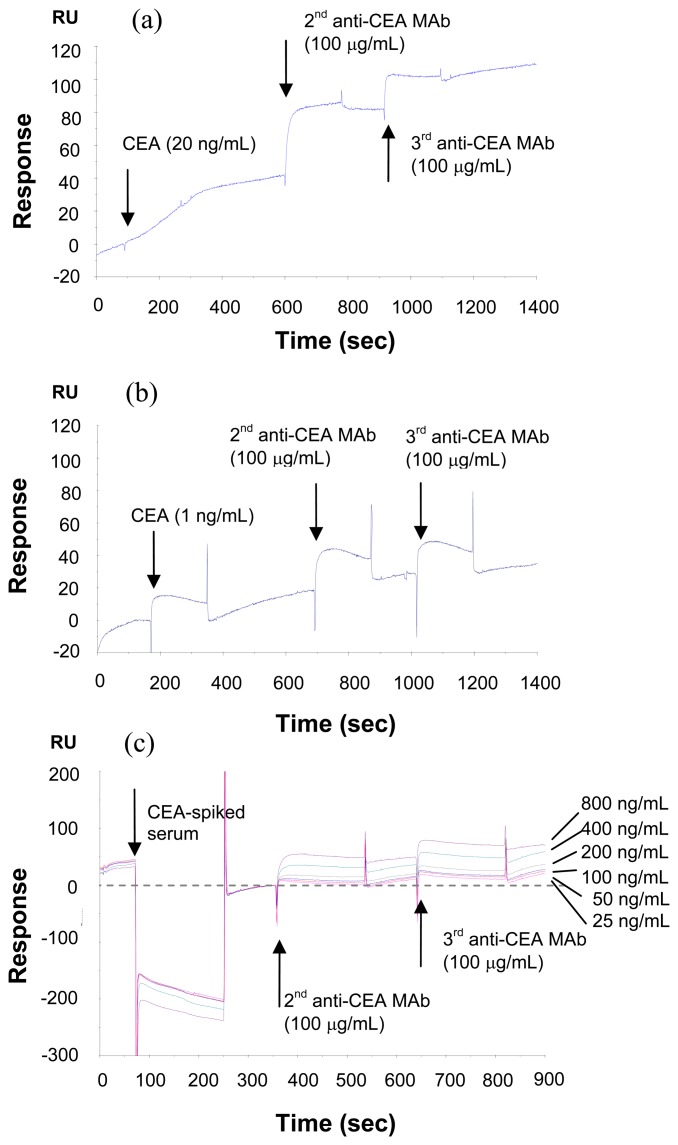
Sandwich assay of 20 ng/mL (a) and 1.0 ng/mL (b) of CEA in HBS buffer, and a series of CEA-spiked serum sample (c).

As an alternative to improve the detection sensitivity of CEA on SPR, a sandwich assay was used to detect CEA antigen with a low concentrations while maintaining the short analysis time of 180 seconds. In this work, three anti-CEA antibodies were selected: RDI detecting as the anchor antibody to be immobilized onto the sensor surface, and RDI coating and anti-CEA from HuChem as the second and third antibody for the sandwich assay. Figure 5(a) shows the sensorgram of detecting 20 ng/mL of CEA in HBS, the antigen, second antibody and third antibody caused a shift of 30 RU, 50 RU and 20 RU, respectively, so the total signal shift is increased to 100 RU, 3.3 times the shift caused by antigen only. Therefore, it can be concluded that the three kinds of antibodies are able to bind to different epitopes of CEA antigen, and the sandwich assay amplifies the original interaction signal significantly.

Furthermore, the sandwich assay-based SPR experiment was conducted for the CEA antigen with a low concentration of 1.0 ng/mL, and the corresponding sensorgram is shown in Figure 5(b). This figure indicates that the otherwise unclear signal change for the antibody-antigen interactions could become visible after the interactions of antigen with the second and third antibodies. Therefore, the sandwich assay is an efficient method for detecting CEA antigens at very low concentrations.

A trial of detection of CEA in human serum was conducted by analyzing human serum spiked with different amount of CEA (25-800 ng/mL). As shown in Figure 5(c), as little as 25 ng/mL of CEA in serum was clearly detectable when serum was diluted to 1:10 with HBS. Though the detection sensitivity may need to be further improved several times for the detection of low ng/mL of CEA in serum, this approach clearly indicates that SPR could become rapid and informative alternative to detect small amount of CEA in serum. It has been reported that the glycosylation pattern of CEA changes upon malignant transformation [20], which indicates that not only detecting CEA but also characterizing glycoforms present on CEA may help specifying the stage of diseases. In this regard, SPR may be a preferred analytical platform due to its ability to amplify responses using carbohydrate- recognizing molecules (e.g. lectins and anti-carbohydrate antibodies) without the need for labeling.

### 2.4. Influence of the sensor chip

All of the above experiments were conducted on the CM5 sensor chip. In fact, for detecting protein- protein interactions, several kinds of sensor chips are applicable based on the properties of proteins. Since the target analyte CEA has a large molecular weight of around 150 KDa, we also used C1 and a self-assembly monolayer of ethylene-glycol terminated alkanethiols as the sensor chip in order to find the best condition to detect CEA at low concentrations.

CM5 is the most versatile and general sensor chip in the BIACORE sensor chip family, where carboxymethylated dextrans are covalently attached to a gold surface as a thick matrix. The molecules could be covalently coupled to the sensor surface via amine, thiol, aldehyde or carboxyl groups. In this work, the amine group of the antibody was covalently coupled to the carboxyl group of carboxymethylated dextran. C1 is also a good option for antibody-antigen interaction, since C1 has a flat layer terminated with a carboxyl group and is suitable for detecting large molecules, such as antibodies or antigens. In addition, we also considered using a self-assembled monolayer of mercaptoalkanoic acid as the material for sensor chips, because they can form a thin layer with the functional carboxyl group on the gold surface. In order to avoid non-specific binding, the mixture of ethylene glycol-terminated mercapto-alkanol, (1-mercapto-11-undecyl)tetra(ethylene glycol) (HSC_11_EG_4_OH) and ethylene glycol-terminated mercapto-alkanoic acid, (1-mercapto-11- undecyl)hexa(ethylene glycol)carboxylic acid (HSC_11_EG_6_OCH_2_COOH) were used in this research and their self-assembly monolayer are abbreviated as EG-SAM in the following discussion.

**Figure 6.**
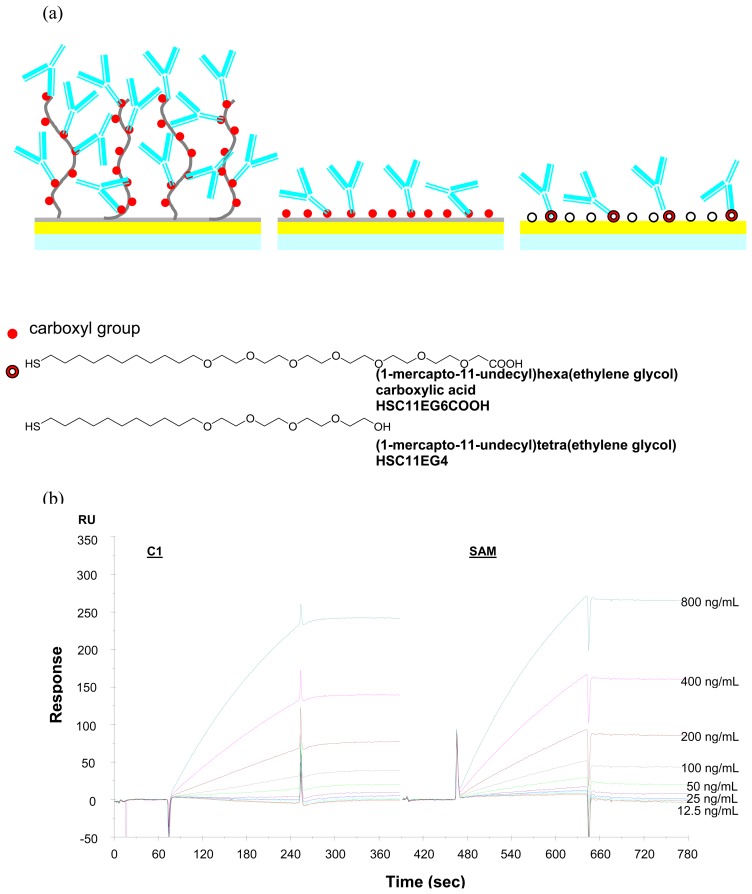
Illustration of the sensor chip CM5, C1 and EG-SAM (a) and the detection of a series of CEA on C1 and EG-SAM (b).

**Figure 7.**
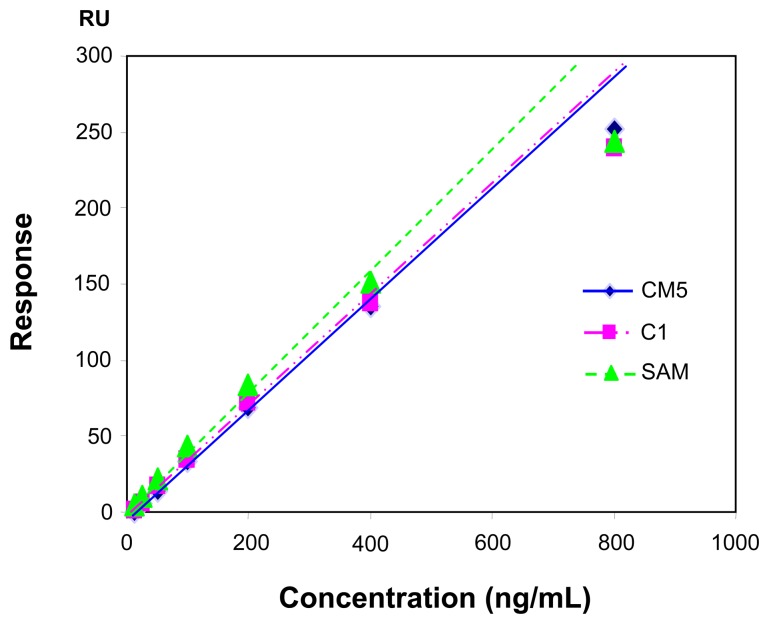
Signal shift as a function of concentration for three kinds of sensor chips; ◊ CM5, □ C1, and Δ SAM.

The immobilization of anti-CEA onto the sensor chip caused a shift of 1,700 RU on C1 and 2500 RU on EG-SAM (the figures are omitted), while the shift was 23,800 RU on CM5 under the same condition. The immobilized amounts of anti-CEA antibody onto C1 and CM5 are much less than that on CM5, most probably because CM5 has a matrix containing more carboxyl group than C1 and EG- SAM. However, the further interactions of antibody-antigen caused quite similar signal shifts on the three kinds of sensor chip CM5, C1 and EG-SAM as shown in sensorgrams in Figures 3 and 6. The response data summarized in Figure 7 gives a more clear view of resonance unit shift with concentrations on different surfaces, namely, there is a linear relationship between resonance unit shift and concentration when the concentration is lower than 400 ng/mL and the shifts on the three kinds of sensor chip are similar to each other.

It is very interesting that the signal shifts for antigen-antibody interactions are very similar, even though the signal shifts for immobilizing antibody are quite different on the three kinds of sensor chips. To understand this phenomenon, the SPR principle and the structures of the sensor chips were examined. The structures of the three kinds of sensor chips are illustrated in Figure 6(a). CM5 has a thick layer of around 100 nm of dextran, while C1 has a thin flat layer on gold and EG-SAM has a layer of 5 nm on gold. According to SPR principle, the SPR signals decay exponentially with the distance of analyte from the surface [21], the change close to the gold film is easier to detect, so the interaction on the top of dextran layer may cause less shift compared to that at the bottom, and there is higher hindrance for the anti-CEA antibody at the bottom to bind with CEA. Therefore, the interactions of antibody and antigen would presumably cause similar signal shift even though there are more antibodies on CM5.

## 3. Experimental

### 3.1. Materials

Carcinoembryoic antigen (CEA) was from Acris Antibody Company, Germany; RDI-CEA-1401 detecting and RDI-CEA-1410 coating anti-CEA antibodies were from Fitzgerald; Anti-CEA antibody is also obtained from Chemicon International. *N*-hydroxysuccinimide (NHS), *N*-ethyl-*N′*-(3-dimethyl- aminopropyl)carbodiimide (EDC), 1.0 M ethanolamine (pH 8.5), anti-mouse rabbit IgG polyclonal antibody, glycine·HCl (pH 1.5), HBS-P buffer (pH 7.4), and 10 mM acetate buffer (pH 5.0) were from GE Healthcare. HSC_11_EG_4_OH and HSC_11_EG_6_OCH_2_COOH were from Prochimio, Poland. The HBS buffer comprised 10 mM HEPES, pH 7.4, 150 mM NaCl, and 0.05% surfactant P20 in distilled water.

### 3.2. Instruments

The BIACORE 2000 SPR was operated at a constant temperature of 20 °C. The fluidic system contains four flow-cells, and one flow-cell is used as a reference to subtract possible non-specific signals and correct for refractive index changes, injection noise, and instrument drift. BIACORE results are expressed in resonance units (RU), which correspond to the resonance angle shift in real time, 1,000 RU is equivalent to 0.1° angle shift. CM5, C1 and CM-Au sensor chips were from BIACORE. CM5 and C1 were used directly for the experiments. CM-Au was used as the substrate for self-assembly. 1mM of HSC_11_EG_4_OH and HSC_11_EG_6_OCH_2_COOH were mixed in ethanol in a molar ratio of 1:2 and self-assembled onto the CM-Au.

### 3.3. Immobolization of anti-CEA antibody

Anti-CEA antibody was immobilized on a sensor chip via anti-mouse IgG polyclonal antibody, via Protein A or Protein G, and through direct covalent binding. The immobilization of anti-CEA antibody via anti-mouse IgG involves several steps. First, the carboxylic groups of the dextran matrix or the SAM layer on a sensor chip were activated by injection of a solution containing 0.05 M NHS and 0.2 M EDC in deionized water. Next, a 100 μL of rabbit anti-mouse IgG polyclonal antibody solution (100 μg/mL in 10 mM acetate buffer pH 5.0) was injected, followed by injecting 100 μL of 1 M ethanolamine pH 8.5 to block remaining NHS-ester groups. Finally, the solution of monoclonal antibody in HBS was injected and allowed to react with anti-mouse IgG polyclonal antibody. Immobilization of anti-CEA antibody via Protein A or Protein G was performed in a similar manner, except the addition of anti-mouse IgG polyclonal antibody is replaced by addition of 100 μg/mL of Protein A or Protein G in 10 mM acetate buffer (pH 5.0) for 14 min.

For covalent immobilization of anti-CEA antibody, the mixture of NHS and EDC, anti-CEA antibodies, and ethanolamine were successively injected over the sensor surface. HBS was used as running buffer; a continuous flow of HBS at 10 μL/min was maintained during the immobilization procedure.

Analyte solutions were then perfused over the antibody-immobilized surface at a flow rate of 20 μL/min for 3 min. Solutions containing different CEA concentrations were prepared in HBS (pH 7.4). Regeneration of the surfaces was performed by pulses of 10 mM glycine HCl (pH 1.5) between each analyte injection.

### 3.4. Signal enhancement using second and third antibodies

Signal amplification was performed by employing a sandwich approach. RDI-CEA-1401 detecting anti-CEA antibody from Fitzgerald was used as the ligand immobilized onto the chip surface, RDI- CEA-1410 coating anti-CEA antibody from Fitzgerald and anti-CEA from Chemicon International were used as the second and third antibodies for the sandwich assay.

## 4. Conclusions

In this work, the performance of a SPR biosensor for the detection of CEA in human serum was evaluated. Based on the finding that the immobilizations of anti-CEA antibody via anti-mouse polyclonal IgGs, Protein A and Protein G were not suitable for the detection of CEA in serum due to the undesired binding with human IgG in serum, anti-CEA antibody was covalently immobilized onto the surface using amine coupling chemistry. Kinetic analysis of the interactions of CEA with immobilized anti-CEA antibody allowed precisely simulating their interactions, which enabled us to optimize the analytical conditions capable of detecting purified CEA at a concentration as low as 6.2 ng/mL. The simulation indicates that this sensitivity will be readily improved by simply increasing the immobilizing amount of anti-CEA antibody and/or extending the interaction time. It was further demonstrated that a sandwich assay, where the resonance signal is amplified by injecting 2^nd^ and 3^rd^ anti-CEA antibodies, can substantially improve the detection sensitivity. SPR with sandwich assay was found to detect purified CEA at a concentration of 1 ng/mL, and CEA in human serum at a concentration of 25 ng/mL when serum was diluted 10-fold.
